# Development and Validation of the Holistic Cognition Scale

**DOI:** 10.3389/fpsyg.2021.551623

**Published:** 2021-09-30

**Authors:** Andrei Alexander Lux, Steven Lee Grover, Stephen Tai Theng Teo

**Affiliations:** ^1^School of Business and Law, Edith Cowan University, Joondalup, WA, Australia; ^2^Department of Management, Macquarie University, Sydney, NSW, Australia

**Keywords:** analytic, holistic, cognition, thinking, scale, measure, culture, cultural differences

## Abstract

This paper introduces a new scale to measure cognitive cultural differences, drawing on the theory of analytic versus holistic thought. Examining culture from a cognitive perspective is a challenge to traditional values-based approaches. Existing measures based on this framework are methodologically problematic and warrant renewal. This paper presents development and validation studies for a new instrument that measures analytic versus holistic cognitive tendencies at the individual level. The scale assesses four previously established dimensions: attention, causality, contradiction, and change. The present work follows well-established scale development protocols and the results show that the 16-item Holistic Cognition Scale (HCS) is a valid and reliable measure of analytic versus holistic thought. Three new studies with four unique samples (*N* = 41; 272; 454; and 454) provide evidence to support the content validity, reliability, and factor structure of the new instrument, as well as its convergent, discriminant, and concurrent validity against comparable constructs. Convergent validity is established against measures of compromise, intuition, complexity, and collectivism; predictive validity is established against Hofstede’s (1980) five cultural value dimensions; and discriminant validity is established using the average variance extracted from a confirmatory factor analysis. The new HCS is an improvement over previous attempts with a balanced number of forward- and reverse-scored items, superior reliability, less redundancy, and stronger factor loadings.

## Introduction

Interest in cross-cultural business research has increased sharply over the last few decades (Boer et al., 2018). Cultural differences shape our values, norms, behavior, emotions, and cognition (Masuda et al., 2020), influencing our work experiences and work-related outcomes. Various motives advance cross-cultural research, including extending or challenging existing theories, testing their generalizability, developing new cultural theories and scales, comparing known effects among existing constructs, and studying multicultural interactions across contexts (for a review, see Gelfand et al., 2017). In an increasingly global and culturally diverse world, better understanding cross-cultural nuances enables us to harness the cultural diversity across and within geographical zones to improve individuals’ quality of life and advance organizational goals.

Traditional explanations for cultural differences in the management literature are based predominantly on values and focus on comparisons between countries (for a review, see Taras et al., 2016). A more contemporary approach to understanding and capturing cultural differences addresses the underlying cognitive processes: examining *how* people think, instead of *what* they think (Nisbett and Miyamoto, 2005). Basic cognitive processes such as inference, perception, causal reasoning, attention, and categorization were assumed to be universally homogeneous by mainstream psychologists throughout the 20th century. More recent research suggests that the habitual use of these cognitive processes varies systematically across global populations (Henrich et al., 2010). Cognitive processes remain malleable well into adult life and people who have been “socialized from birth into different world views and habits of thought” develop distinct cognitive systems, or ways of thinking (Nisbett et al., 2001, p. 291). Two dominant approaches persist and are conceived of in the literature as the theory of analytic versus holistic thought (Nisbett et al., 2001).

Empirical interest in analytic versus holistic cognition is growing with a proliferation of research across a range of psychology domains, including: decision-making (Savani et al., 2017; Li et al., 2018), linguistic abstraction (Klein et al., 2010), consumer behavior (Hossain, 2018), facial emotional recognition (Tanaka et al., 2012; Meaux and Vuilleumier, 2016), and social task performance (Apanovich et al., 2018). Two extant scales measure the construct of analytic versus holistic thought: the Holism scale by Choi et al. (2003) and the Analysis-Holism Scale (AHS) by Choi et al. (2007). The Holism scale represents an initial foray into measurement as part of establishing the theory of analytic versus holistic thought and includes only two dimensions of holistic tendencies. In contrast, the AHS resulted from a concerted scale development initiative comprised of multiple studies.

The AHS (Choi et al., 2007) derives from thorough theoretical integration of several disciplines. The conceptual foundation establishes a clear dimensional structure, which is a critical first step toward creating sound psychometric measures (Schriesheim et al., 1993; Hinkin, 1995). However, concerns persist in the AHS with regards to its low reliability, low factor loadings, cross-loading between dimensions, and discriminant validity. The psychometric issues in the AHS may be driven by including questions (hereafter referred to as “items”) that are highly redundant, that pose double-barreled questions, and the asymmetric number and dispersion of reverse-coded items. The goal of psychometric scale development is to create a valid measure of an underlying construct with a set of reliable and unidimensional items (Clark and Watson, 1995). Therefore the development of a new instrument to measure analytic versus holistic thought may be warranted.

The purpose of creating the Holistic Cognition Scale (HCS) in this paper is to provide a psychometrically strong scale that captures all components of the analytic versus holistic thought construct. The HCS is an entirely new instrument resulting from an original scale development initiative; the HCS does not include or revise any items previously found in the Analysis-Holism Scale (Choi et al., 2007) or the Holism scale (Choi et al., 2003). Developing the HCS in idiomatic English supports its application across a large portion of the globe for which existing instruments may not be suitable. The HCS thereby enables researchers to test existing theories across culturally diverse contexts by investigating the direct, moderating, mediating, and cross-level effects of holistic cognition on known constructs. The following sections outline the origins of the analytic and holistic cognitive systems and then examine the four underlying dimensions identified by Choi et al. (2007). The remainder of the paper is devoted to reporting three sequential scale development studies that demonstrate the validity and psychometric properties of the HCS.

### Cognitive Tools

Between roughly the fifth and second century BCE, Greece and China, in particular, saw considerable advancement in philosophy and moral thought (Jaspers, 1953). The unique circumstances of these two civilizations spurred the simultaneous development of remarkably different social structures. Ancient Chinese society was complex and hierarchical with prescriptive roles and a focus on harmony, whereas ancient Greek society was less complex, characterized by personal agency and an emphasis on debate. Nisbett et al. (2001) argue that the social structure and philosophical ideology in ancient Greece and China directly affected the development of residents’ cognitive frameworks by making certain patterns of interaction more preferable than others. For example, if individuals are encouraged to contend with one another, they establish rules to govern these debates, such as formal logic and the principle of non-contradiction, which would otherwise be of little use to people whose society was based on harmony and compromise (Becker, 1986).

Lévi-Strauss (1966) illustrates such processes by conceiving of people as *bricoleurs* – handymen armed with sets of cognitive tools that they use to engage the quandaries of their daily lives. These tools are the embodiment of a culture’s intellectual history containing their unique theorizations about the world, which are then accepted by the users of these tools (Resnick, 1994). The cognitive system, or toolkit, predominantly used by the ancient Greeks can be broadly labeled as analytic and that of the ancient Chinese as holistic (Nisbett, 1998; Peng and Nisbett, 1999). Individuals are not born as analytic or holistic thinkers, rather their cognitive patterns have historical, philosophical, and sociological origins that render them relatively distinct (Choi et al., 2003).

Nisbett et al. (2001) explain that contemporary thinkers are similarly armed with cognitive toolkits and which tools they reach for most often is a function of their culturally embedded metaphysical and epistemological frame. Beliefs about the world determine individuals’ approach toward knowledge, which in turn shapes their habitual preference for certain cognitive toolkits. Individuals have access to both analytic and holistic cognitive approaches, but a dominant and socially reinforced preference emerges. For example, holistic thinkers may appreciate the qualities of a focal object but typically tend toward a broader grasp of its nature in relation to the salient context. The analytic and holistic toolkits are distinct and diametrically opposed: for instance, focusing on a single object precludes a simultaneous focus on the context, likewise a steady linear perception of change precludes a more turbulent cyclical perspective. Consistent with this logic, we follow the theoretical foundations established by Nisbett et al. (2001) and develop the HCS with a unidimensional structure. Analytic and holistic cognition thereby form polar ends of a single dimension of sociocultural cognitive orientation. Scale item wording is directed toward the holistic frame, so that higher HCS scores indicate more holistic cognition and lower scores indicate more analytic cognition. The unidimensional approach to the HCS is therefore consistent with the theoretical structure advanced by Choi et al. (2007).

Because such deeply ingrained cultural elements evolve very slowly over time (Beugelsdijk et al., 2015), these two cognitive patterns have endured to the modern era and “find their counterparts among contemporary peoples” (Nisbett et al., 2001, p. 292). These ideas are encapsulated in the literature as analytic versus holistic thought (Nisbett and Miyamoto, 2005) and provide a valuable new perspective for examining cultural variations across and within national borders. The following sections first define analytic and holistic thought and then examine the various dimensions included in scale development.

### Definitions of Thought

Cross-cultural and cognitive science research generally accepts that Western thought is particularly analytic and that Eastern thought is more holistic (Spencer-Rodgers et al., 2010). In their seminal article Nisbett et al. (2001) define analytic thought as:

Involving detachment of the object from its context, a tendency to focus on attributes of the object to assign it to categories, and a preference for using rules about the categories to explain and predict the object’s behavior. Inferences rest in part on the practice of decontextualizing structure from content, the use of formal logic, and avoidance of contradiction (p. 293).

In contrast, they define holistic thought as:

Involving orientation to the context or field as a whole, including attention to relationships between a focal object and the field, and a preference for explaining and predicting events on the basis of such relationships… there is an emphasis on change, a recognition of contradiction… and a search for the “middle way” between opposing propositions (p. 293).

Building on this theoretical foundation Choi et al. (2007) categorize the key differences between analytic and holistic thought in terms of attention, causality, contradiction, and perceptions of change.

### Attention: Object vs. Field

Attention explains where people focus mentally in the external environment. Contemporary Westerners predominantly focus on primary objects and rely on categorization and rules to conceptually organize their environment (Nisbett and Miyamoto, 2005), while Easterners instead focus on the relationships between objects and within the environment. For example Chiu (1972) finds that Easterners presented with a picture of a man, a woman, and a child are more likely to group together the woman and the child because “the mother takes care of the baby,” whereas Westerners usually select the man and the woman, because “they are both adults” (p. 237).

### Causality: Dispositionism vs. Interactionism

Dispositionism is an approach to explaining behavior and events by examining the internal characteristics and motivations of individuals. An interactionist explanation, on the other hand, focuses on the contextual circumstances and the relationships between people (Benforado and Hanson, 2008). Easterners tend to adopt an interactionist approach and consider all aspects of the world to be interconnected (Masuda and Nisbett, 2001; Spina et al., 2010). Westerners predominantly demonstrate a dispositional approach by attributing events and behavior to the primary actors (Choi et al., 2007) and perceiving the world as many independent objects (Hansen, 1983).

### Contradiction: Formal Logic vs. Dialectics

The third dimension of analytic versus holistic cognition concerns the differences between individuals’ approach to discourse. Analytic thinkers use deductive syllogism and the principle of non-contradiction (Liu, 1974; Becker, 1986), while holistic thinkers use a dialectic approach that involves reconciling, accepting, and transcending contradiction (Lloyd, 1990; Nisbett et al., 2001). Easterners are more comfortable with contradiction and tend to pursue compromise when presented with two seemingly incompatible propositions by finding value in both arguments (Spencer-Rodgers and Peng, 2017). In contrast, when Westerners encounter two contradictory statements they typically examine both sides and choose one of the two alternatives (Davis et al., 2000; Choi et al., 2007).

### Perception of Change: Linear vs. Cyclic

Easterners see the world as a complex web of interrelated elements and therefore believe that all such elements exist in a perpetual state of flux as a result of the myriad ongoing interactions (Choi et al., 2007). Such holistic thinkers generally anticipate frequent cyclical change of greater magnitude when formulating predictions about the future (Ferris et al., 2018). Because Westerners perceive objects as largely independent from one another their essence is neither affected by external factors nor does it change dramatically over time (Choi et al., 2007). Therefore, when analytic thinkers speculate about the future they tend to expect less turbulent change and gradual linear progress based on their past experiences (Peng and Nisbett, 1999; Ji et al., 2001).

### Current Prevalence

In the present day, analytic thought prevails primarily in the West, spanning across Europe, North America, Canada, Australia, and New Zealand. Holistic thought is predominant throughout the East, including East and Southeast Asia, Japan, and Korea, as well as in India and the Middle East (Spencer-Rodgers et al., 2010; Ronen and Shenkar, 2013). Together these regions contain the majority of the current global population, including almost all of the developed and more than half of the newly industrialized countries. Widespread coverage of the foremost commercial zones around the world positions the construct well as a lens for investigating how cultural differences interact with business processes and outcomes.

The following sections report three studies that develop and validate the Holistic Cognition Scale (HCS). We follow the scale development guidelines provided by Worthington and Whittaker (2006): new items are first subject to expert review (Study 1), followed by an exploratory factor analysis (EFA; Study 2), and confirmatory factor analysis (CFA; Study 3) using different samples at each stage.

## Study 1: Scale Development and Content Validation

Contemporary scale development techniques utilize quantitative methods to reduce the subjectivity of item assessments (Schriesheim et al., 1993; Hinkin and Tracey, 1999). These techniques can help refine scales and improve content validity before subsequent research initiatives invest into potentially problematic measures (Schriesheim et al., 1999). The present study adopts the four-dimensional framework of analytic versus holistic thought established by Choi et al. (2007) and begins the scale development process by generating a pool of items and testing their content validity.

### Method

#### Sample

A total of 276 participants were invited to the study and 41 respondents returned complete data (response rate = 14.9%) which is an appropriate sample size for a content validation study (Hinkin and Tracey, 1999). Respondents included 19 doctoral students and 22 academic faculty members from Management Departments in six medium-sized universities across New Zealand. The sample was comprised of 56.1% males and the mean age was 43.6 years. Following Schriesheim et al. (1999) we selected doctoral students and academic staff versed in management literature for this study because they are generally familiar with business research terminology and possess sufficient intellect to understand the construct definitions. The scale is intended for use with businesspeople and management subject matter experts are therefore apt to ensure that the scale items are both fit for purpose and suitable for the target audience.

#### Procedure

Based on a review of the literature and consistent with Choi et al. (2007) we defined each aspect of the four analytic and holistic thought dimensions (see Table 1).

**TABLE 1:** Study 1 – definitions of the Holistic Cognition Scale (HCS) dimensions.

Attention *(analytic)*	Analytic thinkers tend to focus their attention on the primary object, detaching it from the context and assigning it to categories based on its attributes, because they see the world as comprised of numerous independent pieces.
*(holistic)*	Holistic thinkers orient toward the context as a whole, focusing on the relationships between objects, because they believe that parts exist only within wholes, to which they have inseparable relations.

Causality *(analytic)*	Analytic thinkers understand cause and effect by focusing on the internal dispositions, characteristics, or motivations of individuals, because they believe that people are mostly independent and events occur as a result of individual peoples’ behavior.
*(holistic)*	Holistic thinkers understand cause and effect by examining the contextual circumstances and interactions between people, because they believe that all things are interconnected and events occur as a result of the complex relations between various people and within the environment.

Contradiction *(analytic)*	The analytic approach to discussion assumes that all propositions must either be true or false, and that they cannot be both at the same time; when faced with two contradictory statements, the most plausible alternative is chosen and the least plausible is rejected.
*(holistic)*	The holistic approach to discussion involves reconciling, accepting, and transcending contradiction, understanding that even opposing ideas can both be true; when faced with two contradictory statements, a compromise is sought by finding value in both arguments.

Change *(analytic)*	Analytic thinkers see change as a linear progression which moves through incremental and permanent adjustments; when speculating about the future they anticipate gradual linear progress based on their past experiences.
*(holistic)*	Holistic thinkers see the world as in a constant state of change, which moves in cycles that transform each element into its opposite and then back into itself again; when speculating about the future they anticipate frequent cyclical change of greater magnitude.

We followed the principles of scale item writing set out by Clark and Watson (1995, p. 312) to create an initial pool of 55 survey items using a deductive approach based on these four construct definitions. Thus, the weaker items could be identified by subsequent analysis and removed from the emerging scale (DeVellis, 2012). All of the items were written as declarative statements for use with a Likert-type scale response format and the wording targeted an eight grade reading level, as is appropriate for instruments intended for use with the working age adult population (Flesch, 1949). We took particular care to ensure that both the holistic and analytic aspects of each dimension were well represented in this preliminary item pool (Clark and Watson, 1995) and varied the item wording to sufficiently “sample all possible contents” of the theory (Loevinger, 1957, p. 659).

The initial set of items was distributed to the respondents as an online survey via an email link. Participation was anonymous and voluntary, without incentive, and all respondents provided informed consent to participate. In addition to the demographic questions the survey contained eight main sections one for each of the eight definitions. Participants were provided the construct definitions one at a time at the start of each section and were instructed to rate the extent to which each item captured that corresponding definition. A seven-point Likert-type scale was provided to rate item relevance as either: 1 = Not at all, 2 = Slightly, 3 = Somewhat, 4 = Fairly, 5 = Moderately, 6 = Very well, or 7 = Completely. The definitions were included at the top of each page for reference.

### Analysis and Results

We used a quantitative approach to identify appropriate items for retention so as to avoid the pitfalls of subjective judgment (Hinkin and Tracey, 1999). The technique was based on the work by Schriesheim et al. (1993) and involved comparing the items’ mean ratings – which are an indication of how well they captured the corresponding conceptual dimension according to the expert panel of respondents. Following McGraw and Wong (1996) we first calculated a two-way random effects inter-class correlation to test for absolute agreement of mean ratings among the sample of raters. The results indicate excellent inter-rater reliability: ICC (2) = 0.93, 95% CI = [0.89, 0.95] (Portney and Watkins, 2009). We therefore ranked the items in each section according to their mean rating and retained the three highest-rated items from each dimension for further testing. Examination of median item ratings supported the decisions with broadly consistent results.

Where two theoretically identical items with slightly different wording ranked among the three top items across that dimension we retained only one to avoid any unnecessary redundancy (see Clark and Watson, 1995). In addition, several items within the holistic sections were particularly well rated but not among the top three and addressed different aspects of those dimensions. We likewise retained these for further testing to ensure that the entire breadth of the construct would be adequately covered. In total, we retained 28 items from the initial pool which demonstrated adequate content validity according to our expert panel of respondents and were thus congruent with the theoretical definitions of the analytic versus holistic thought construct.

## Study 2: Testing the Factor Structure and Validity

Having established the theoretical content validity of the HCS during the previous study the next step in the scale development process is to field-test its dimensionality (Hinkin, 1998). DeVellis (2012) notes that this is also an opportune time to test a new scale’s convergent validity by including several well-established measures for comparison. As such, we included items from the Rahim Organizational Conflict Inventory II (ROCI-II; Rahim, 1983), the Rational-Experiential Inventory (REI; Epstein et al., 1996), and the Attributional Complexity Scale (ACS; Fletcher et al., 1986) to establish convergent validity comparisons. These scales sought to gauge respondents’ attitudes toward compromise, intuition, and complexity, respectively – concepts similar to the holistic aspects of the HCS. We also included a measure of Collectivism (COL; Oyserman et al., 2002) to represent the cultural values typically associated with Eastern respondents. We anticipated weak (0.1–0.3) positive correlations with all four constructs as evidence of the scale’s convergent validity (for correlation strength thresholds, see Dancey and Reidy, 2004, pp. 170–171). Discriminant validity was established using Fornell and Larcker’s (1981) average variance extracted (AVE) test which dictates that the square root of the AVE for each construct must be larger than its absolute correlation with any other construct.

### Method

#### Sample

The final sample consisted of 306 undergraduate student participants from Business Schools across several Australian and New Zealand universities. Cases with missing data were deleted list-wise to preserve fidelity, resulting in 272 usable responses. This was considered to be adequate as a sample of 200–300 participants is recommended for the purposes of structural analysis in scale development (Guadagnoli and Velicer, 1988; Clark and Watson, 1995). Of the 272 respondents, 162 (59.6%) were female and the average age for the sample was 21.9 years old (SD = 7.1). The data obtained from such a sample would not accurately represent the intended future target population of business people but are sufficient to investigate the psychometric adequacy and validity of the scale (DeVellis, 2012).

#### Measures

Participants were recruited during class time. Their participation was voluntary, anonymous, and without incentive. The paper-based survey collected demographic information and contained 82 items in total which included the 28 retained items of the HCS as well as the following four criterion measures. All self-report instruments were scored on the same Likert-type scale with seven response categories: 1 = Completely disagree, 2 = Disagree, 3 = Somewhat disagree, 4 = Neither, 5 = Somewhat agree, 6 = Agree, or 7 = Completely Agree. Scores were calculated using the mean of all scale items.

##### Rahim Organizational Conflict Inventory II

Rahim (1983) developed this scale to explore five different approaches to handling interpersonal conflict: integrating, avoiding, dominating, obliging, and compromising. The four items that capture individuals’ tendency to compromise were extracted from the ROCI-II and included in the present study to provide a convergent validity comparison for the HCS.

##### Rational-Experiential Inventory

Epstein et al. (1996) developed the REI to measure individuals’ preferences for rational thought or intuition. The REI contains 10 items which are split evenly into two dimensions called “need for cognition” and “faith in intuition”; we extracted the five pertaining to intuition for use in the present study.

##### Attributional Complexity Scale

Fletcher et al. (1986) developed the ACS to measure the level of complexity in individuals’ attributional processes. The ACS contains 28 items assessing seven sub-dimensions of attributional complexity of which we deemed three as relevant for the purposes of the present study: preference for complex, complex-internal, and complex-external explanations. The 12 items addressing these dimensions were extracted for use here.

##### Individualism and Collectivism

Individualism is characterized by a preference for autonomy and focus on personal advancement, whereas collectivism emphasizes the mutual benefit of common purpose and prioritizes in-group harmony. Oyserman et al. (2002) used meta-analysis to synthesize one coherent instrument with 15 items that captured the most “consensual operationalization of IND and COL across researchers” (p. 10). In contrast to Individualism and Collectivism, which are conceived of here as two distinct dimensions (for a review, see Li and Aksoy, 2007), the HCS is a unidimensional construct with analytic and holistic thought at opposite ends of a single continuum. We therefore included the eight items pertaining to Collectivism to serve as the most appropriate comparable construct for holistic thought.

#### Analysis and Results

We follow the recommendations by Cortina (1993) and first conduct an EFA on the 28 content-validated items using principal components analysis (PCA) to ascertain the appropriate number of underlying dimensions and to narrow down the item selection. SPSS v24 software was used to perform the analysis. Following the steps set out by Clark and Watson (2019) we eliminated items that cross-loaded (over 0.40) on multiple dimensions as well as those that loaded on the wrong dimension. As a result 16 of the initial 28 items were dropped and 12 retained. We ran the EFA again on only the remaining 12 items and the first five eigenvalues were 3.35, 1.41, 1.32, 1.16, and 0.90, indicating that it was appropriate to extract four factors (Kaiser, 1960; Cattell, 1966) which together accounted for 60.3% of the total variance. Examining the slope of a scree test (see Gorsuch, 1983) largely supports the extraction of four factors with a notable plateau around the fourth factor.

We then used rotation to produce a “simple structure” where item loadings range between zero and absolute 1; items that load closer to one are important in the interpretation of the factor and items that load closer to zero are unimportant (Bryant and Yarnold, 1995). Attaining a simple structure is essential to factor analysis (Kline, 1994). Orthogonal rotation is used when factors are uncorrelated (Byrne, 2005) for which Gorsuch (1983) recommends the Varimax method. The HCS dimensions are theoretically distinct: for example, attention and contradiction are unrelated constructs. We therefore proceed with Varimax orthogonal rotation for interpretation, sorting items by dimension and highest value (see Table 2 for the resulting 12 items and their factor loadings).

**TABLE 2:** Study 2 – exploratory factor analysis of the 12-item HCS.

Dimensions – Items	1	2	3	4
**Attention**				
1. Where you put an ornament is just as important as the ornament itself.	**0.74**	0.17	–0.12	0.09
2. In a painting, the background is just as important as the main object.	**0.72**	–0.03	0.16	0.12
3. It is impossible to understand the pieces without considering the whole picture.	**0.70**	0.17	0.21	0.22
**Causality**				
4. Events occur as a result of individuals’ choices and behavior. ^*r*^	–0.04	**0.81**	0.06	0.16
5. Each individual person is only responsible for his/her own actions. ^*r*^	0.20	**0.74**	–0.03	–0.01
6. People’s behavior is best understood by carefully examining their personal characteristics and internal motivations. ^*r*^	0.12	**0.63**	0.22	0.12
**Contradiction**				
7. Even two seemingly contradictory ideas can each yield something valuable.	0.10	0.02	**0.77**	–0.02
8. In any given situation, what it means to do the right thing depends on who you ask.	0.14	0.13	**0.76**	0.16
9. Things can be both ugly and beautiful at the same time.	–0.02	0.07	**0.63**	0.17
**Change**				
10. It is useful to make future forecasts based on present situations. ^*r*^	0.15	0.09	–0.03	**0.87**
11. An honest man can be expected to stay honest in the future. ^*r*^	0.17	0.05	0.14	**0.77**
12. The course of human history is best described as gradual linear progress. ^*r*^	0.10	0.16	0.24	**0.71**

*N = 272. ^r^ Indicates reverse-coded items. Extraction: Principal Component Analysis. Rotation: Varimax with Kaiser Normalization.*

We next tested the reliability of these 12 items by estimating internal consistency using Cronbach’s coefficient alpha, composite reliability, and average variance extracted (AVE). Scale reliability refers to an instruments ability to measure the intended construct consistently and precisely. Internal consistency estimates, such as Cronbach’s alpha, are the preferred test of psychometric scale reliability (Hogan et al., 2000). The 12-item HCS exceeded the 0.70 threshold for acceptable alpha and composite reliability (Hair et al., 2010; DeVellis, 2012) yet fell short of the recommended 0.50 threshold for AVE. However, psychometricians caution against relying solely on reliability indices to establish scale homogeneity (see Green et al., 1977; Boyle, 1991; Cortina, 1993) because they can be artificially inflated by retaining highly inter-correlated items which reduces scale validity – i.e., the classic attenuation paradox in psychometric theory (see Loevinger, 1954, 1957; Briggs and Cheek, 1986). Therefore, we also calculated the average inter-item correlations (AIC) which should range between 0.15 and 0.50 and nearer to 0.20 for broad higher order constructs such as analytic versus holistic thought (Clark and Watson, 1995, p. 316). See Table 3 for the reliability analysis results which indicate that the HCS demonstrates sufficient internal consistency, and therefore, reliability. By eliminating equivalent items (survey questions) we have ensured that the HCS captures the breadth of this construct without narrowing its measurement focus through redundant items that serve only to inflate reliability indices.

**TABLE 3:** Study 2 – reliability analysis of the 12-item HCS.

Scale	# Items	α (95% CI)	CR	AVE	MSV	AIC
Holistic Cognition Scale	12	0.73 (0.69, 0.78)	0.74	0.42	–	0.21
– Attention	3	0.59 (0.50, 0.67)	0.63	0.37	0.28	0.35
– Causality	3	0.61 (0.52, 0.68)	0.62	0.35	0.19	0.34
– Contradiction	3	0.60 (0.51, 0.67)	0.63	0.37	0.17	0.34
– Change	3	0.70 (0.63, 0.76)	0.75	0.51	0.28	0.49

*N = 272. α = Cronbach’s Coefficient Alpha. CI, confidence interval; CR, composite reliability; AVE, average variance extracted; MSV, maximum shared variance; AIC, average inter-item correlation.*

We assessed the scale’s convergent validity by calculating Pearson’s correlations between the 12-item HCS and the four criterion measures (see Table 4 for the results). Each of the criterion measures demonstrated acceptable psychometric properties and reliability to proceed with analysis (see Table 4 for coefficient alphas in brackets on the diagonal). As anticipated, the HCS correlated positively with the ROCI-II measure of compromise, the REI measure of intuition, the ACS scale of cognitive complexity, and with Collectivistic cultural values. Taken together these results demonstrate the convergent validity of the HCS – that the latent construct captured by this instrument is in fact related to theoretically comparable constructs that are already well-established in the field. The correlations were weak, ranging between 0.19 and 0.28, suggesting that these constructs are relatively similar but not so much so that they could supplant the HCS. We tested the scale’s discriminant validity with Fornell and Larcker’s (1981) AVE test which confirmed that the HCS captures a unique construct that is statistically distinct from existing instruments (see Table 5 for the results). Therefore, the HCS is not simply an iteration of established measures, rather it captures a novel perspective.

**TABLE 4:** Study 2 – correlation analysis of the 12-item HCS.

Variable	Mean	SD	1	2	3	4	5
1. HCS (12-item)	2.56	0.62	(0.73)				
2. Compromise	2.95	0.94	0.25^**^	(0.85)			
3. Intuition	2.99	0.99	0.28^**^	–0.00	(0.83)		
4. Complexity	3.60	1.02	0.19^**^	0.10	0.02	(0.79)	
5. Collectivism	2.58	0.79	0.24^**^	0.42^**^	0.13^*^	–0.13	(0.71)

N = 272, *p < 0.05, **p < 0.01. Coefficient alphas are in brackets on the diagonal.

**TABLE 5:** Study 2 – validity analysis of the 12-item HCS.

Variable	CR	AVE	MSV	1	2	3	4	5
1. HCS (12-item)	0.74	0.42	0.19	***0.65***				
2. Compromise	0.85	0.59	0.20	0.43	***0.77***			
3. Intuition	0.83	0.50	0.17	0.41	0.01	***0.71***		
4. Complexity	0.79	0.39	0.07	0.26	0.10	0.04	***0.63***	
5. Collectivism	0.76	0.40	0.20	0.32	0.45	0.11	–0.10	***0.63***

*N = 272. CR, composite reliability; AVE, average variance extracted; MSV, maximum shared variance. Bold, italicized numbers denote Fornell and Larcker (1981) AVE test.*

The results of the present study indicate that the 12-item HCS is a valid and sufficiently reliable measure of analytic versus holistic thought. However, the reliability indices for the individual dimensions returned relatively low scores (coefficient alphas between 0.59 and 0.70) and the AVE for the overall scale was below the recommended 0.50 threshold, suggesting that further revision and evaluation is warranted.

#### Pilot Study

Having identified the kinds of items that worked well the next phase of the scale development process includes adding new questions to each of the four dimensions to refine the emerging instrument. We conducted a brief pilot study using a series of focus groups with a selection of subject matter experts from the first study to generate several additional items for each of the four dimensions. Participants were provided with the 12 items that worked well in the second study and engaged in unstructured open dialogue to co-create additional survey questions, resulting in eight new scale items. Several minor adjustments were also made to existing items based on participants’ feedback: for example, “an honest man…” was changed to “an honest person…” to avoid gendered language. Psychometric instrument development is an iterative process (Cronbach, 1984), and therefore, the following study builds on the present results.

## Study 3: Refining for Reliability and Confirming Validity

The third study used two larger samples of businesspeople (*N* = 454 and *N* = 454) to refine the scale for reliability, verify the dimensional structure using confirmatory factor analysis (CFA), and then test for concurrent validity by regressing the HCS on a well-established measure of cultural differences. To establish the concurrent validity of the HCS we compared it against Hofstede’s (1980) five-dimensional framework of cultural values which is a dominant approach in the cross-cultural management domain (Beugelsdijk et al., 2017). Ronen and Shenkar (2013) categorize each of the five cultural values identified by Hofstede (1980) as being either predominantly “Eastern” or “Western” in nature. People with Eastern cultural values are largely collectivistic and feminine, they accept a greater disparity in power, prefer to avoid ambiguity, and orient toward the long-term. Those with Western cultural values are primarily individualistic and masculine, they tolerate less power distance, are more comfortable with uncertainty, and prioritize the short-term (see Ronen and Shenkar, 2013, p. 885). As a cognitive theory that derives from historical, philosophical, and sociological roots, we theorized that the HCS would also predict respondents’ tendency to exhibit certain cultural values that are typically associated with such origins. Because the HCS is phrased to capture respondents’ preference for holistic thought we expected that it would correlate negatively with Western cultural values such as individualism and masculinity and positively with Eastern cultural values such as high power distance, uncertainty avoidance, and long-term orientation.

### Method

#### Sample

The study participants were employed adults currently living in Australia and a total of 908 respondents returned completed surveys. Ages ranged from 18 to 75 years old (mean = 45.4, SD = 11.9). Most of the respondents were born in Australia (74.9%) and spoke English as their first language (87.6%). The participants’ were predominantly female (63.1%), full-time employees (64.9%) of large organizations (52.7%). A broad range of private and public sectors were well represented in the data. The 908 respondents were randomized into two unique samples without replacement (*N* = 454 in each sample). No substantive demographic differences were evident between the two samples. Sample 1 was used to conduct the EFA and Sample 2 was used for the CFA, reliability, and concurrent validity analysis.

#### Measures

The web-based survey included the 12-item HCS, the eight new scale items generated through focus groups in the pilot study, and 20 items from the 2008 Values Survey Module (Hofstede et al., 2010) to establish the concurrent validity of the HCS. The same seven-point Likert-type scales were used to rate items as in Study 2 and means were calculated to determine scale scores.

### Analysis and Results

We first conducted an exploratory factor analysis using the expanded set of HCS items to assess the number of underlying dimensions. Items that cross-loaded (over 0.40) on multiple dimensions or loaded on to the wrong dimension were eliminated, leaving 16 HCS items in total. The first five eigenvalues were 4.64, 2.11, 1.45, 1.20, and 0.87; therefore, indicating that it was appropriate to again extract four factors (Kaiser, 1960; Cattell, 1966), which accounted for 58.8% of the total variance. The procedure was repeated with Varimax orthogonal rotation for interpretation; the items sorted first by dimension and then by highest value (see Table 6 for the remaining 16 items and their respective factor loadings).

**TABLE 6:** Study 3 – exploratory factor analysis of the 16-item HCS.

Dimensions – Items	1	2	3	4
**Attention**				
1. It is impossible to understand the pieces without considering the whole picture.	**0.79**	–0.05	0.12	0.15
2. It makes more sense to study things in their natural context rather than in isolation.	**0.74**	0.01	0.09	0.05
3. In a painting, the background is just as important as the main object.	**0.71**	0.22	0.28	0.16
4. Relationships between people are more important than the individuals themselves.	**0.63**	0.18	0.25	–0.04
**Causality**				
5. People are mostly independent of one another. ^*r*^	0.23	**0.79**	0.18	0.18
6. Events only occur as a result of individuals’ choices and behavior. ^*r*^	–0.11	**0.74**	0.17	0.16
7. Each individual person is only responsible for his/her own actions. ^*r*^	0.13	**0.71**	–0.05	0.08
8. People usually end up doing what they want to, irrespective of norms or expectations. ^*r*^	0.05	**0.69**	0.14	0.13
**Contradiction**				
9. Something can be both ugly and beautiful at the same time.	0.32	0.06	**0.82**	0.07
10. In any given situation, what it means to do the right thing depends on who you ask.	0.14	0.07	**0.74**	0.01
11. It is better to reach a compromise than to argue your point of view.	0.14	0.10	**0.69**	0.01
12. Even two seemingly contradictory ideas can each yield something valuable.	0.10	0.23	**0.60**	0.29
**Change**				
13. An honest person can be expected to stay honest in the future. ^*r*^	0.09	–0.02	–0.04	**0.79**
14. A person’s true nature may evolve but it does not change dramatically over time. ^*r*^	0.06	0.13	–0.01	**0.75**
15. It is useful to make future projections based on the present situation. ^*r*^	0.04	0.34	0.20	**0.75**
16. The course of human history is best described as gradual linear progress. ^*r*^	0.09	0.21	0.18	**0.50**

*Sample 1, *N* = 454. ^*r*^ Indicates reverse-coded items. Extraction: Principal Component Analysis. Rotation: Varimax with Kaiser Normalization.*

The resulting 16 items were subject to CFA using maximum likelihood estimation and the AMOS v24 software package to verify the scale’s dimensional structure. The maximum likelihood method requires normally distributed continuous variables and cannot be used if these assumptions are violated (Li, 2016). However, ordinal variables recorded on 7-point Likert-type scales can also be safely treated as continuous for multivariate analysis. An examination of the underlying data revealed absolute skewness values less than 3 and absolute kurtosis values less than 10 which indicate sufficient normality (Kline, 2015). We therefore proceed with maximum likelihood estimation and compared three different models against each other for best fit: (a) a single-factor model with all 16 items loading on to the same factor, (b) a first-order model with the items loading on to four corresponding factors (attention, causality, contradiction, and change), and (c) a second-order model with the four first-order factors loading on to a single latent second-order factor, which represents the theoretically derived structure of analytic versus holistic thought.

**FIGURE 1:**
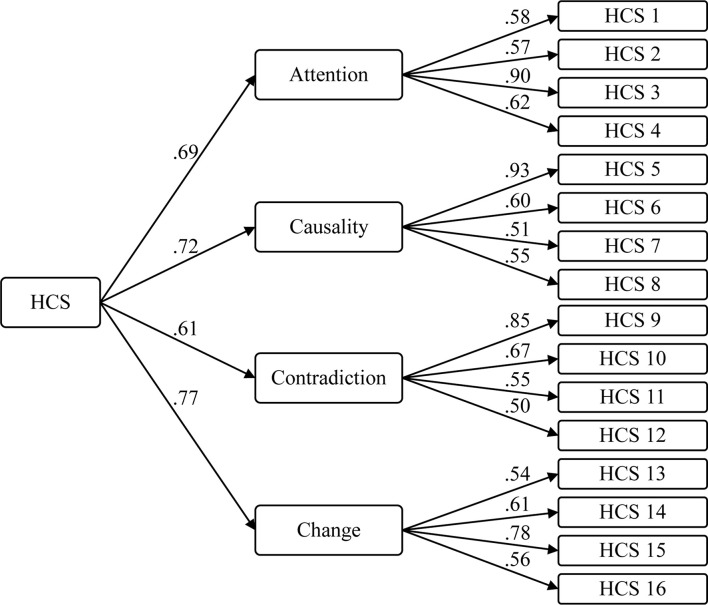
Study 3 – confirmatory factor analysis of the 16-item HCS. Sample 2, *N* = 454.

Model fit was assessed with chi-square (*χ*^2^), ratio of chi-squared to the degrees of freedom (*χ*^2^/*df*), goodness of fit (GFI), comparative fit (CFI) and normative fit (NFI) indices, as well as the root mean square error of approximation (RMSEA). The results indicate that the second order model (c) demonstrated the best relative fit and satisfactory fit indices: GFI, CFI, and NFI values over 0.90 (Byrne, 1994; Hu and Bentler, 1999), *χ*^2^/*df* ratio less than 5.0 (Carmines and McIver, 1981; Schumacker and Lomax, 2004), and an RMSEA less than 0.08 (Browne and Cudeck, 1993) or ideally, less than 0.05 (Steiger, 1990). Overall, these results confirm that the factor structure of the final 16-item HCS corresponds with the theory of analytic versus holistic thought (see Table 7 for the model fit results and Figure 1 for the CFA diagram).

**TABLE 7:** Study 3 – structural equation model analysis of the 16-item HCS.

Model	*χ*^2^ (*df*)	*χ*^2^/*df*	GFI	CFI	NFI	RMSEA (90% CI)
(a) Single-factor model	244.39^*^ (84)	2.91	0.94	0.93	0.90	0.07 (0.06, 0.07)
(b) First-order model	253.29^*^ (94)	2.70	0.93	0.93	0.89	0.06 (0.05, 0.07)
(c) Second-order model	213.91^*^ (99)	2.35	0.95	0.95	0.91	0.06 (0.05, 0.06)

Sample 2, *N* = 454. * Indicates significant chi-square test, *p* < 0.05.

The internal consistency of the scale was re-established using the same techniques as previously; first by estimating the coefficient alpha, composite reliability (CR), and average variance extracted (AVE), and then by calculating the average inter-item correlations (AIC). Acceptable reliability indices include coefficient alpha and CR above 0.70 (Hair et al., 2010; DeVellis, 2012), AVE above 0.50 (Fornell and Larcker, 1981), and AIC between 0.15 and 0.50 (Briggs and Cheek, 1986; Clark and Watson, 1995). See Table 8 for the reliability analysis results which indicate that the final 16-item version of the HCS is a sufficiently reliable measure of analytic versus holistic thought. The scale is a significant improvement over the earlier 12-item variant with each dimension reporting an alpha over 0.70 and higher scores for the overall scale across all of the reliability indices.

**TABLE 8:** Study 3 – reliability analysis of the 16-item HCS.

Scale	# Items	α (95% CI)	CR	AVE	MSV	AIC
Holistic Cognition Scale	16	0.83 (0.81, 0.85)	0.80	0.50	–	0.26
– Attention	4	0.76 (0.72,0.80)	0.77	0.46	0.31	0.46
– Causality	4	0.70 (0.65, 0.74)	0.75	0.44	0.37	0.41
– Contradiction	4	0.70 (0.65, 0.74)	0.74	0.43	0.31	0.40
– Change	4	0.71 (0.67, 0.75)	0.71	0.39	0.37	0.40

*Sample 2, *N* = 454. α = Cronbach’s Coefficient Alpha. CI, confidence interval; CR, composite reliability; AVE, average variance extracted; MSV, maximum shared variance; AIC, average inter-item correlation.*

Having established the psychometric adequacy of the 16-item HCS, the next phase of the development process involves testing the scale’s criterion-related validity. There are two types of criterion validity, concurrent and predictive; the difference rests solely in the time at which each measure is administered. The present study included Hofstede’s (1980) five cultural value dimensions to serve as suitable concurrent validity criterion for this analysis. Hofstede’s (1980) dimensions demonstrated marginally acceptable psychometric properties with reliability estimates around the minimum threshold (see Table 9 for coefficient alphas in brackets on the diagonal). Pearson’s correlations revealed significant negative relations between the HCS and individualism (*r* = −0.36, *p* < 0.01) and masculinity (*r* = −0.42, *p* < 0.01), and positive relations with power distance (*r* = 0.44, *p* < 0.01), uncertainty avoidance (*r* = 0.39, *p* < 0.01), and long-term orientation (*r* = 0.53, *p* < 0.01). See Table 9 for the results. The directionality of these relations corresponds with our predictions since higher HCS scores indicate more holistic cognitive tendencies and stand in contrast to Western values.

**TABLE 9:** Study 3 – correlation analysis of the 16-item HCS.

Variable	Mean	SD	1	2	3	4	5	6
1. HCS (16-item)	2.93	0.63	(0.83)					
2. Individualism	5.75	0.91	–0.36^**^	(0.69)				
3. Masculinity	5.28	0.96	–0.42^**^	0.68^**^	(0.72)			
4. Power distance	2.61	0.86	0.44^**^	–0.63^**^	–0.63^**^	(0.75)		
5. Uncertainty avoid.	2.80	0.88	0.39^**^	–0.26^**^	–0.25^**^	0.34^**^	(0.66)	
6. Long-term orient.	2.56	0.83	0.53^**^	–0.38^**^	–0.34^**^	0.35^**^	0.52^**^	(0.69)

Sample 2, *N* = 454, **p < 0.01. Coefficient alphas are in brackets on the diagonal.

The moderate magnitude of the correlations between the HCS and Hofstede’s cultural value dimensions suggests that the cognitive focus of the HCS is different from the traditional values-based approach. We reconfirmed the refined scale’s discriminant validity using Fornell and Larcker’s (1981) AVE test which requires that the square root of the scale’s AVE is larger than its error-corrected correlations with any other construct (Venaik et al., 2005). See Table 10 for the results that establish that the 16-item HCS is statistically distinct from individualism, masculinity, power distance, and uncertainty avoidance. Some overlap was evident between the HCS and long-term orientation which may have been a function of the marginally acceptable reliability of Hofstede’s instrument. Establishing such discriminant validity is important because it demonstrates that the HCS captures a unique perspective and is difficult to supplant with existing measures.

**TABLE 10:** Study 3 – validity analysis of the 16-item HCS.

Variable	CR	AVE	MSV	1	2	3	4	5	6
1. HCS (16-item)	0.80	0.50	0.65	***0.71***					
2. Individualism	0.68	0.42	1.08	–0.59	***0.65***				
3. Masculinity	0.72	0.41	1.08	–0.60	1.04	***0.64***			
4. Power distance	0.74	0.50	0.87	0.58	–0.93	–0.93	***0***.**71**		
5. Uncertainty avoid.	0.72	0.48	0.45	0.54	–0.29	–0.28	0.32	***0***.***69***	
6. Long-term orient.	0.70	0.44	0.65	0.81	–0.53	–0.47	0.46	0.67	***0***.***66***

*Sample 2, *N* = 454. CR, composite reliability; AVE, average variance extracted; MSV, maximum shared variance. Bold, italicized numbers denote Fornell and Larcker (1981) AVE test.*

The results from the third study replicate and support the findings from the second study using a larger and more representative sample. Exploratory and confirmatory factor analyses confirm the second-order four-dimensional structure. The pattern of correlations observed between the HCS and theoretically comparable constructs establishes the scale’s convergent validity and the AVE test supports its discriminant validity. The results of the present scale development studies suggest that the 16-item HCS has acceptable psychometric properties and is therefore suitable for use in organizational and cross-cultural research. The Flesch (1949) readability score (56) and Flesch–Kincaid grade level (8.4) indicate that the HCS items will be easily understood by working-age respondents. The HCS is designed for use with seven-point Likert-type agreement scales and HCS scores are derived by calculating the mean across all scale items: higher scores indicate more holistic cognitive tendencies and lower scores indicate more analytic cognitive tendencies.

## Discussion

This paper contributes to cross-cultural research by developing a valid new measure of individual cultural differences using a cognitive approach based on the theory of analytic versus holistic thought (Nisbett et al., 2001). Three studies are presented that successfully develop items and established their reliability and validity. The HCS is advantaged with a balanced number of forward- and reverse-scored items, superior reliability, stronger factor loadings, and less redundancy than previous attempts such as the AHS. The new HCS thereby opens the door to future research in both cross-cultural psychology and business disciplines such as organizational behavior, human resource management, marketing, entrepreneurship, and international business, as well as more broadly across social science domains including politics, sociology, education, and communication studies. Scholars working in these fields can deploy the HCS across culturally diverse contexts to test the effects of holistic cognition on salient latent constructs to apply, extend, and challenge existing theories, or develop new theoretical models.

Most organizational research on cultural differences examines shared and relatively stable cultural *values* (Taras et al., 2009; Caprar et al., 2015) using either Hofstede’s (1980) original values framework or latter refinements (e.g., Schwartz, 1994; House et al., 2004). Cultural values are acquired by virtue of individuals’ membership and engagement with society. However, global mobility promotes cultural heterogeneity as diverse people move between cities, nations, and societal spheres, picking up and sharing their cultural outlook along the way. While societies collectively co-construct what they believe to be important and preferable, individuals simultaneously develop cognitive schema that govern how they engage the world.

The present research attempts to address the limitations of traditional values-based approaches (cf. Taras et al., 2016) with an instrument specifically targeted at such individual level cognitive phenomena that is better suited to capture the complex nuances of modern peoples’ cultural differences. The HCS thereby creates an opportunity to shift gears in cross-cultural research by examining the cognitive schema that people use to engage the world, studying *how* they think at the individual level, instead of *what* they think at various levels of aggregate abstraction.

### Advantages Over Previous Scales

The HCS developed in this paper creates a window of opportunity by improving on several methodological issues that persist in previous attempts at measuring analytic–holistic orientation. Specifically, the present scale is advantaged over the 24-item AHS (Choi et al., 2007) by addressing four major concerns related to: (1) item wording; (2) reliability; (3) factor loading; and (4) discriminant validity.

The AHS includes highly redundant items that artificially increase coefficient alpha but undermine the scale’s content validity by narrowing how much of the latent construct is captured by the instrument (Boyle, 1991). Some of the AHS items also pose double-barreled questions, for example: “Any phenomenon has numerous numbers of causes, although some of the causes are not known.” Choi et al. (2007) explain that the AHS items were originally written in Korean and it is not clear whether a robust back-translation method (Brislin, 1980) was used to convert the items into English. To avoid such issues the items comprising the HCS were screened for any complex, double-barreled, or ambiguous statements to ensure a consistent interpretation across a range of respondents. The HCS is further advantaged with varied item wording that addresses the edges of the analytic versus holistic thought construct (Loevinger, 1957; Briggs and Cheek, 1986). The AHS only includes six reverse-coded items out of 24 which may contribute to survey response bias (for a review, see Smith et al., 2016) and acquiescence bias (Smith, 2004). We therefore follow the recommendations proposed by Weijters et al. (2013) to include an equal number of forward- and reverse-coded items in the HCS that mitigate acquiescence bias issues. The reverse-coded items are not simply negatively worded (e.g., using “*not*” to alter semantic direction), which Barnette (2000) condemns as a practice of questionable utility. Following Barnette’s (2000) recommendations, the reverse coded HCS items are instead phrased to positively sample the opposite polarity of the construct (i.e., the analytic aspect instead of the holistic). Such an approach is preferable to the logical reversal of otherwise identical items (for reviews, see Rorer, 1965; Segura and González-Romá, 2003).

The HCS demonstrates superior reliability across the overall scale (α = 0.83) and within each dimension (α = 0.76, 0.70, 0.70, and 0.71) above the 0.80 overall scale threshold recommended by Clark and Watson (1995) for any new instrument. Setting the standard for development above the accepted 0.70 minimum (Nunnally, 1978) allows for some sample variance in future research applications without jeopardizing the scale’s reliability. In contrast, the scale development studies that introduce the AHS report three alpha coefficients (0.74, 0.73, and 0.68) for the final 24-item scale (Choi et al., 2007). The coefficient alpha estimate is a function of scale length and increases with additional items (Briggs and Cheek, 1986), which should be evident in a relatively long instrument such as the AHS. When we consider the inflationary effect of redundant items and scale length the results reported by Choi et al. (2007) raise considerable reliability concerns for the AHS. These issues persist at the dimensional level with three out of the four AHS dimensions reporting suboptimal alpha scores (α = 0.69, 0.58, and 0.56).

The factor structure of the HCS is supported by confirmatory factor analysis (see Table 7). The results demonstrate that the second-order model with four dimensions is the best fit to the data and corresponds with the theoretical model of analytic versus holistic thought. The unidimensionality of the HCS is further evidenced by the scale’s average inter-item correlation (0.26) which is within the optimal range to balance breadth with fidelity (Briggs and Cheek, 1986). The HCS is therefore an improvement over the AHS which reports relatively low factor loadings that are 0.56 on average and range from 0.19 to 0.76 (see Table 1, Choi et al., 2007, p. 694). Seven of the items load below 0.50 and several reveal non-trivial cross-loading on other factors (e.g., 40, 0.35, etc.). These results signal a lack of cohesion within each of the AHS dimensions and that the comprising items are not capturing a single clearly defined latent construct. In comparison, the HCS items demonstrate stronger factor loadings which are 0.71 on average and range from 0.50 to 0.82 (see Table 6); none are below 0.50 and none cross-load above 0.35 with other factors.

Fornell and Larcker (1981) propose a superior way to test for discriminant validity using average variance extracted from a CFA. The results of the AVE test reveal that the HCS is statistically distinct from measures of compromise, intuition, and complexity, as well as Hofstede’s (1980) five cultural value dimensions. The interpretation of these results supports the discriminant validity of the HCS. In contrast, Choi et al. (2007) report that the AHS is discriminantly valid because it does not correlate significantly with measures of individualism–collectivism or independent–interdependent self-construal. However, it is problematic that the AHS is unrelated to collectivism in light of the regional, historic, philosophical, and sociological overlap between collectivistic cultures and the origins of holistic cognition. According to the analytic versus holistic cognition theory people who emanate from more collectivistic societies should have developed more holistic cognitive systems (Nisbett et al., 2001). While these constructs are theoretically distinct, it would make more sense to observe a significant positive correlation of a small magnitude, such as that between the HCS and collectivism (*r* = 0.24, *p* < 0.01).

The HCS treats analytic and holistic thought as polar ends of a single construct: higher scores indicate more holistic cognitive tendencies and lower scores indicate more analytic cognitive tendencies. The HCS demonstrates weak positive relations with measures of compromise, intuition, and complexity, which suggest that the HCS is working as intended among comparable constructs. For instance, holistic cognition is characterized by a dialectic approach to discourse that promotes compromise (Nisbett et al., 2001); holistic thinkers tend to process a larger array of contextual information that requires a more complex cognitive schema (Nisbett and Miyamoto, 2005); and intuition is described as an automatic and holistic decision-making process in contrast to a more intentional analytic approach (Epstein et al., 1996). Therefore, significant positive correlations of a weak magnitude between the HCS and such associated constructs offer evidence of the scale’s convergent validity.

The HCS is also positively correlated with Eastern cultural values, including high power distance, uncertainty avoidance, and long-term orientation, and negatively correlated with Western cultural values, including individualism and masculinity (Ronen and Shenkar, 2013). The direction of these relations supports the predictive validity of the scale because we can hypothesize that higher HCS scores will predict respondents’ Eastern cultural values that share its historical and sociological roots. The moderate magnitude of the correlations between the HCS and Hofstede’s cultural value dimensions suggests that the cognitive approach of the HCS is distinct from traditional values-based conceptions and cannot be supplanted with existing measures.

Campbell and Fiske (1959, p. 82) argue that the correlations between theoretically identical instruments should be significant and “sufficiently large” to support the convergent validity of a new scale. Clark and Watson (2019) moderate this requirement by considering how conceptually similar the convergent validity constructs are to the new scale: for example, in Study 2 we correlate the HCS with compromise, intuition, complexity, and collectivism; these constructs are somewhat related but far from identical to analytic–holistic thought. Therefore it is appropriate to see significant correlations of a weak magnitude as evidence of convergent validity. In Study 3 we correlate the HCS with Hofstede’s (1980) five cultural values, which conceptually resemble analytic–holistic thought much more closely and therefore we see significant correlations of a larger magnitude as evidence that the HCS is behaving as expected against comparable constructs.

### Limitations

Scales with unequal numbers of forward- and reverse-scored items are susceptible to acquiescence bias (Smith et al., 2016) – that is, some respondents are more likely to agree to survey questions irrespective of the item content (Paulhus, 1991). A disproportionate number of positively framed items will therefore yield an artificially inflated score and distort effect sizes. The prevalence of acquiescence bias varies between cultural contexts and such instruments incur significant validity issues in cross-cultural research (Smith, 2004). The HCS is therefore advantaged with a balanced number of forward- and reverse-scored items. However, the forward-scored items are concentrated in two dimensions (attention, contradiction) and the reverse-scored items are concentrated in the other two dimensions (causality, change). Because the HCS is a latent higher-order construct represented by four dimensions the dispersion of forward and reverse-coded items throughout the instrument has negligible effect on overall validity. Grouping items scored in the same direction also serves to reduce careless responding errors (Weijters et al., 2013). Individual dimensions should be used with caution and a gestalt approach including the entire scale is recommended.

The present studies introduce the HCS and offer evidence supporting its psychometric properties sufficient for the initial development of a novel instrument. Convergent validity is established against comparable constructs, including compromise, intuition, complexity, and collectivism. Predictive validity is established against Hofstede’s (1980) five cultural value dimensions: individualism, masculinity, power distance, uncertainty avoidance, and short- vs. long-term orientation. Discriminant validity is established against these same constructs using the Fornell and Larcker (1981) AVE test. However, the above list of pertinent covariates is non-exhaustive and countless other relevant constructs and instruments exist in the field. For example, it would also be useful to compare the HCS against the MSG Thinking Style Inventory (Sternberg and Wagner, 1991) and the Self-Construal Scale (Singelis, 1994). The predictive power of the HCS could be tested with behavior-based evaluations, such as the Cognitive Styles Analysis (Riding, 1991), or with cognitive tasks that capture analytic–holistic thinking. Predictive validity could also be tested by comparing the HCS scores of different ethnic groups or nationalities (e.g., Asian vs. White, Hong Kong vs. Australia, etc.).

Psychometric scale development is a well-documented iterative process, built around a series of stepwise refinements that maintain the overall integrity of the emerging instrument (Cronbach, 1984). The resulting scale is typically distinct from earlier versions: desirably so, as evidence of improvement. The tests conducted earlier in the development process thus cannot logically apply to the final scale. Following these guidelines we refined the HCS items between studies and while not strictly necessary it may be prudent to retest the final 16-item HCS for content validity (Study 1) and convergent validity (Study 2) against the same comparable instruments used previously. The unidimensionality of the HCS could also be tested with a polytomous Rasch model (Christensen et al., 2002). Lastly, we recommend that cross-cultural comparative researchers test the measurement and structural equivalence of the HCS to ensure that the instrument measures analytic and holistic cognition in the same way across different populations (Byrne and Van de Vijver, 2010). Overall, the initial evidence presented here is encouraging, but conclusions drawn about the validity of the HCS must be tempered by the limitations of the research design and further work may be warranted.

### Future Research

By offering a psychometrically sound measure of analytic and holistic thought we seek to stimulate researchers to pursue three major ambitions. First, to begin investigating the antecedents and consequences of analytic and holistic cognition. Understanding how holistic cognitive differences affect, and are affected by, other relevant concepts will embed the construct within the broader research context. Such investigations should include individual differences as well as interpersonal, group, and organizational processes. Second, to explore whether known relations are mediated or moderated by analytic and holistic cognition. Some antecedents may make an either holistic or analytic approach more salient, thereby altering the effect on outcome variables, or rather, more holistic or analytic thinkers may respond differently to the same stimulus. Third, to engage in translations of the HCS. The scale was created in English with items that represent the edges of the construct to create a complete measurement. The instrument is ready for immediate use with any English-speaking population. However, cross-cultural research often requires the use of multiple languages and we therefore encourage researchers to use a back-translation method that maintains the complete spirit of the HCS.

### Conclusion

Examining culture from a cognitive perspective is an advance from the traditional values-based approach that has dominated management literature. We contribute by providing a valid measure of analytic versus holistic thought that addresses methodological concerns with current offerings and opens the door for future research. This fresh perspective can help provide answers to a nearly limitless array of research questions and should prove fruitful for many years to come. With the HCS we offer a way forward – moving beyond countries and beyond values toward more meaningful distinctions that capture why certain groups of people think and behave differently.

## Data Availability Statement

The raw data supporting the conclusions of this article will be made available by the authors, without undue reservation.

## Ethics Statement

The studies reported here were conducted according to internationally accepted ethical standards and were reviewed and approved by the University of Otago Human Ethics Committee, reference numbers D15/226, 15/116, and 16/019 respectively. All subjects gave written informed consent to participate in the studies in accordance with the Declaration of Helsinki.

## Author Contributions

AL conceived the idea and devised the project in collaboration with SG. AL collected the data, analyzed the results, and wrote the first manuscript draft. SG supported the development of scale items and provided extensive manuscript revision. ST supervised the analytical methods and refined the presentation of the findings. All authors discussed the results and contributed to the final manuscript.

## Conflict of Interest

The authors declare that the research was conducted in the absence of any commercial or financial relationships that could be construed as a potential conflict of interest.

## Publisher’s Note

All claims expressed in this article are solely those of the authors and do not necessarily represent those of their affiliated organizations, or those of the publisher, the editors and the reviewers. Any product that may be evaluated in this article, or claim that may be made by its manufacturer, is not guaranteed or endorsed by the publisher.
